# Immunological memory to blood-stage malaria infection is controlled by the histamine releasing factor (HRF) of the parasite

**DOI:** 10.1038/s41598-017-09684-2

**Published:** 2017-08-22

**Authors:** Claudia Demarta-Gatsi, Roger Peronet, Leanna Smith, Sabine Thiberge, Robert Ménard, Salaheddine Mécheri

**Affiliations:** 1 Institut Pasteur, Unité de Biologie des Interactions Hôte Parasites, Paris, F-75015 France; 2 CNRS ERL9195, Paris, F-75015 France; 3 INSERM U1201, Paris, F-75015 France; 4 Institut Pasteur, Unité de Biologie et Génétique du Paludisme, F-75015 Paris, France

## Abstract

While most subunit malaria vaccines provide only limited efficacy, pre-erythrocytic and erythrocytic genetically attenuated parasites (GAP) have been shown to confer complete sterilizing immunity. We recently generated a *Plasmodium berghei* (*Pb*NK65) parasite that lacks a secreted factor, the histamine releasing factor (HRF) (*Pb*NK65 *hrf*Δ), and induces in infected mice a self-resolving blood stage infection accompanied by a long lasting immunity. Here, we explore the immunological mechanisms underlying the anti-parasite protective properties of the mutant *Pb*NK65 *hrf*Δ and demonstrate that in addition to an up-regulation of IL-6 production, CD4^+^ but not CD8^+^ T effector lymphocytes are indispensable for the clearance of malaria infection. Maintenance of T cell-associated protection is associated with the reduction in CD4^+^PD-1^+^ and CD8^+^PD-1^+^ T cell numbers. A higher number of central and effector memory B cells in mutant-infected mice also plays a pivotal role in protection. Importantly, we also demonstrate that prior infection with WT parasites followed by a drug cure does not prevent the induction of *Pb*NK65 *hrf*Δ-induced protection, suggesting that such protection in humans may be efficient even in individuals that have been infected and who repeatedly received antimalarial drugs.

## Introduction

In spite of continued efforts to control the disease, malaria remains a major health problem in many regions of the world, especially sub-Saharan Africa. An effective malaria vaccine would be a valuable tool to reduce the burden of the disease and possibly achieve its elimination. However, the complex biological make-up of *Plasmodium* ^1, 2^ and the many strategies that the parasite has developed to outmanoeuvre the host immune response^3^ make the development of a malaria vaccine a difficult task.

Studies in humans and rodents have shown that the control of blood-stage *Plasmodium* infections depends on both humoral and cellular immune responses^4^. Adaptive immunity is initiated when IFN-γ secreted by CD4^+^ T cells induces optimal activation of cytotoxic CD8^+^ T cells^5^, B cell class-switching^6, 7^ and inflammatory monocytes in the bone marrow to migrate into the spleen^8^ and express FcγRI on macrophages enhancing their phagocytic abilities^9^. These mechanisms generate a robust splenic immune response to the parasite, including prominent germinal centre formation and generation of a memory B and CD4^+^ T cells, as well as plasma cells and protective antibodies^10^. However, other mechanisms oppose the role of T cells in the development of anti-malaria immunity. Indeed, one of the characteristic features of malaria infection is the inability to generate an acquired protective immunity, suggesting that memory T cells develop inadequately or their maintenance is not ensured. Interestingly, recent research has shown an upregulation of inhibitory receptors such as programmed death 1 (PD-1) during malaria blood-stage infections in humans and rodents^11–13^, which could be one reason for the lack of lasting immunity against malaria via lymphocyte exhaustion^14^. Alternative mechanisms may also operate, such as the diversity and the large number of antigens overwhelming the immune system.

Several types of vaccines against blood-stage parasites have been devised. Subunit-based vaccines can induce some degree of protection in laboratory animal models and induce antibody responses able to inhibit *P. falciparum* development *in vitro* ^15^. However, limited efficacy, antigenic diversity and polymorphism represent important obstacles in the development of an efficient subunit vaccine. Immunization with live parasites, which induce immune responses to a broad range of parasite antigens, appears to show greater efficacy.

Various types of live erythrocytic vaccines have been developed. Immunization with limited amounts of erythrocytes infected by the WT *P. falciparum*, followed by drug cure after 3 to 4 parasite developmental cycles, has allowed for protecting 4 of 5 human subjects^16^. Additionally, genetically modified parasites with an altered replication capacity have been shown to lead to self resolving infections leading to potent and long lasting protection against both pre-erythrocytic (sporozoites)^17–21^ and erythrocytic (infected red blood cells)^22–24^ challenges. However, these studies provided only limited information regarding the immunological mechanisms that confer protection.

We recently reported on a *P. berghei* mutant lacking the gene encoding the histamine releasing factor (*hrf*) (*Pb*NK65 *hrf*Δ)^25^, which was found to cause a self-resolving blood-stage infection and induce strong protection. HRF belongs to a class of proteins called translationally controlled tumour proteins (TCTP) homologs. HRF/TCTPs were first described as P21, Q23, and P23 by different teams and are highly conserved multifunctional ubiquitous proteins found in eukaryotes, including *Plasmodium falciparum*, and were reported to act as anti-apoptotic factors, and promote allergies^26–28^ through the release of histamine^29^. *Pf*HRF has been identified in the serum of mildly and severely *P. falciparum* infected Malawian children^29^ and shown to have *in vitro* histamine releasing activity^29^. Moreover, increased levels of histamine in plasma and tissue were associated with the severity of disease in human infected with *P. falciparum* and in several animal models of infections with *Plasmodium* ^30–32^. Indeed, histamine-deficient mice were found to be highly resistant to severe malaria^33^.

Our most recent work showed that lack of HRF causes an IL-6 increase, which boosts T and B cell responses to resolve infection via opsonized parasite-mediated phagocytosis giving rise ultimately to a cross-stage, cross-species and lasting immunity. In the present work, we further explore the molecular basis of protection conferred by this mutant.

## Results

### HRF plays a critical role in parasite development both at the pre-erythrocytic and erythrocytic stages

**Figure 1:**
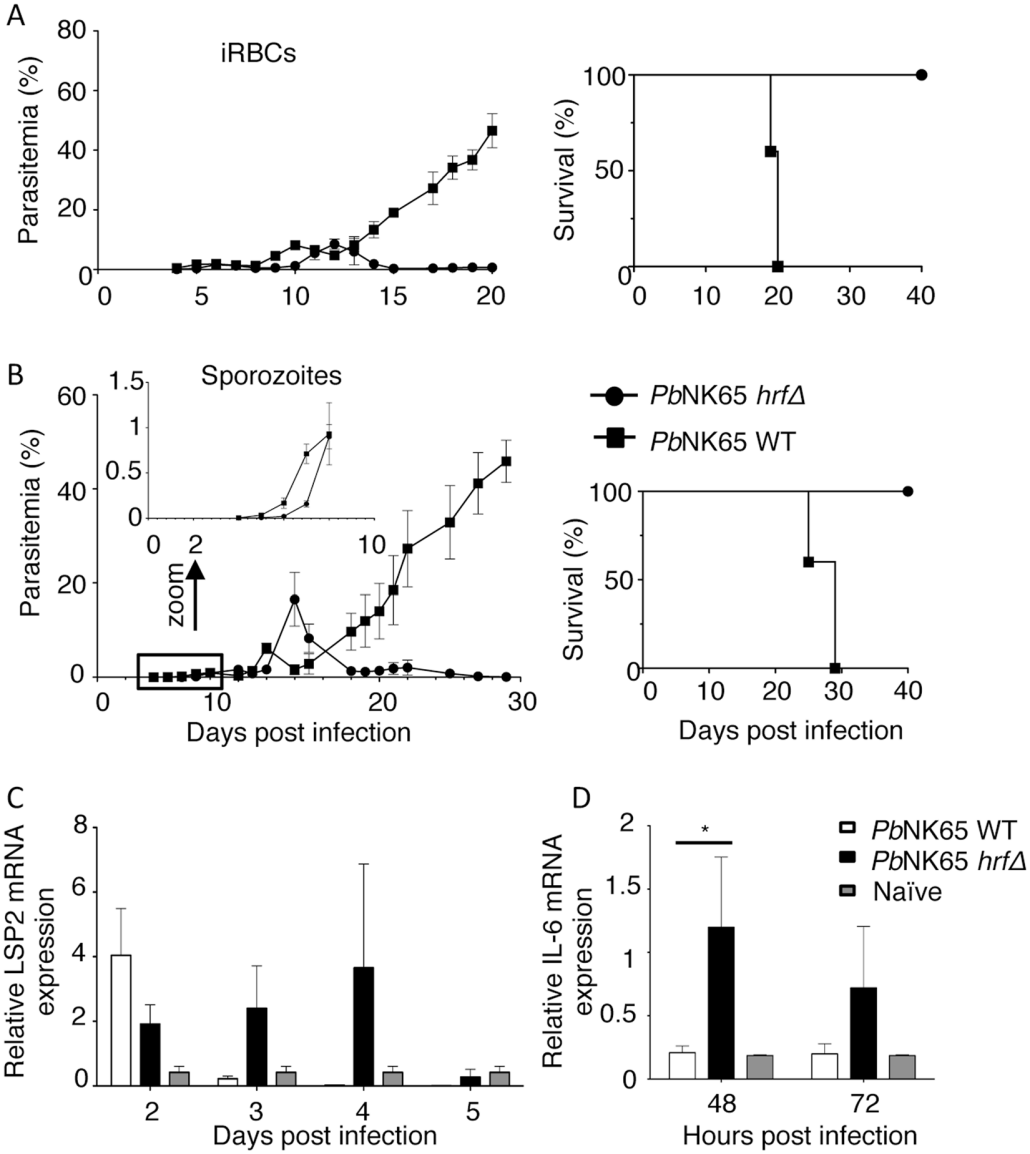
Marked differences in parasitaemia between WT and *Pb*NK65 *hrf*Δ-infected mice during blood stage development. Blood-stage parasitemia and survival (Kaplan-Meier survival plots) of C57BL/6 mice after (**A**) i.p. injection of 10^5^ iRBCs or (**B**) i.v. injection of 10^3^ isolated sporozoites of WT or *Pb*NK65 *hrf*Δ parasites were measured at indicated time points. After infection with WT or *Pb*NK65 *hrf*Δ sporozoites, livers were collected at indicated time points and RT-qPCR analysis were used to measure (**C**) the kinetics of parasite load using the liver stage specific LSP-2 marker expression relative to the parasite control gene HSP70 and (**D**) IL-6 expression using IL-6 mRNA expression relative to mouse HPRT mRNA levels. Error bars, SEM. Data are representative of two independent experiments with 5 mice per group. (*p < 0.05; Mann Whitney test).

As demonstrated earlier^25^, inoculation of mice with *Pb*NK65-*hrf*Δ infected red blood cells (iRBCs) resulted, after an initial phase of normal parasite development, in a self-resolved infection leading to a long lasting protection (Fig. 1A) against challenge with WT parasites^25^ (Supplementary Fig. 1A). We first tested whether HRF might play a role during progression of *Pb*NK65-*hrf*Δ sporozoites *in vivo*. Infection with *Pb*NK65-*hrf*Δ sporozoites resulted in an initial delayed development of the parasite in the liver, followed by a complete clearance of blood-stage infection at around day 17 p.i. (Fig. 1B), a similar outcome as in mice inoculated with blood-stage parasites (Fig. 1A). To assess whether clearance of the parasites after sporozoite inoculation also resulted in protection against a parasite challenge, mice were reinfected at day 36 with *Pb*NK65 WT iRBCs. As shown in Supplementary Fig. 1B, no blood-stage parasite development was observed, when inoculation of naïve mice with WT iRBCs at day 36 used as controls resulted in development of parasitemia and death around day 15 p.i. (day 50 p.i. in the figure). This indicated that self-resolved infection following inoculation by sporozoites or iRBCs of the mutant elicited similar protection.

To analyse liver-stage development *in vivo*, mice were injected with sporozoites intravenously (i.v.) and liver samples collected at 48, 72, 96 or 120 h p.i. were subjected to qRT-PCR analysis of parasite LSP2 RNA (Fig. 1C). At 48 h p.i., the *Pb*NK65-*hrf*Δ parasite load was ∼2 times lower than that of the WT. At 72 hpi., WT parasites were undetectable while the amounts of *Pb*NK65-*hrf*Δ parasites had risen and reached at 96 hpi to levels similar to those of the WT at 48 h p.i. At 120 h p.i., both WT and *Pb*NK65-*hrf*Δ parasites were undetectable in the liver. Therefore, RT-PCR analysis in the liver indicated a > 48 h delay in the completion of the pre-erythrocytic phase. This phenotype is similar to another *hrf* mutant made previously in a distinct *Plasmodium berghei* strain, *Pb*ANKA (*Pb*ANKA-*hrf*Δ)^34^. One of the immunological features observed previously with the *Pb*ANKA-*hrf*Δ model, in contrast to the WT parasite, was an early rise of IL-6 in the liver^34^. Here we assessed the levels of IL-6 expression and found that at 48 hpi, IL-6 transcripts were the sole among those tested that were differentially expressed between infections with WT and *Pb*NK65-*hrf*Δ parasites, being increased six-fold in *Pb*NK65-*hrf*Δ-infected liver samples (Fig. 1D). Therefore the increase in IL-6 production accounts for mutant parasite clearance in both *Pb*ANKA and *Pb*NK65 backgrounds. Since similar phenotypes were obtained with sporozoites and iRBCs, the following investigation was carried out using iRBCs to explore the mechanisms by which HRF modulates the T cell and B cell immune responses.

### Prior exposure to WT parasite followed by drug treatment does not hamper *Pb*NK65-*hrf*Δ-induced parasite clearance and immune protection

**Figure 2:**
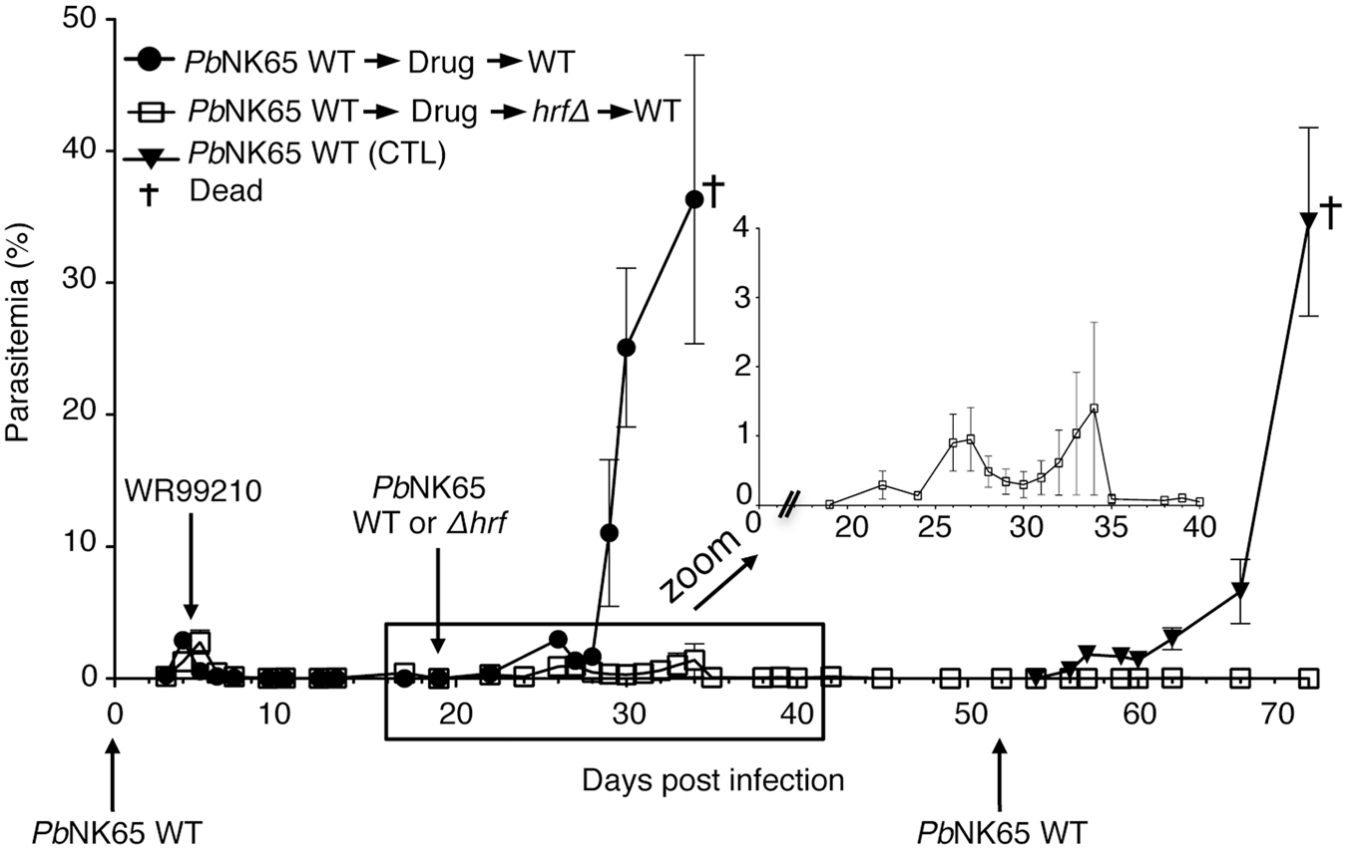
Prior exposure to WT parasite followed by drug treatment does not hamper *Pb*NK65-*hrf*Δ-induced parasite clearance and immune protection. Blood-stage parasitemia of C57BL/6 mice after i.p. injection of 10^5^ WT iRBCs and treated at day 4 p.i. with 6 mg/kg WR99210 by subcutaneous injections for three consecutive days. At day 19 p.i. mice that have eliminated the parasite from blood stream after drug treatment were infected with either with 10^5^ WT or *Pb*NK65 *hrf*Δ iRBCs. Mice infected with *Pb*NK65 *hrf*Δ parasites were subsequently, after parasite elimination from blood stream, challenged at day 52 p.i. with 10^5^ WT iRBCs. Parasite development was measured over several days by flow cytometry. Error bars, SEM. Data are representative of three independent experiments with 5 mice per group.

Results obtained from mice infected firstly with *Pb*NK65-*hrf*Δ parasites and, upon the complete clearance of *Pb*NK65-*hrf*Δ parasites, challenged with either *Pb*NK65, *Pb*ANKA or *Py*YM WT lethal parasites demonstrated that the sterile protection conferred by this genetically attenuated parasite (GAP) was long-lasting in a species- and stage-transcendent manner^25^. One potential important limitation of such an approach for human vaccination, however, is that prior infection resolved by antimalarial chemotherapy might impede the subsequent mounting of effective anti-parasite immunity, as would be the case in endemic areas. To address this issue in our rodent model, mice were first infected with 10^5^ *Pb*NK65 WT iRBCs and, when parasitemia reached ~2%, treated for three consecutive days with 6 mg/kg WR99210 by subcutaneous injections. Once parasites were completely eliminated from the blood stream, at day 19 p.i., mice were infected with 10^5^ WT or with *Pb*NK65-*hrf*Δ iRBCs (Fig. 2). In the group of mice that received WT parasites, parasitemia progressed normally and mice died around day 16 p.i. In contrast, in mice that received *Pb*NK65-*hrf*Δ, parasites displayed slow and limited development and were ultimately eliminated around day 15 p.i. from the peripheral blood (see zoomed inset in Fig. 2). To assess whether a single infection with the mutant parasite was enough to develop a long-lasting immune response, mice that received WT parasites followed by drug treatment and *Pb*NK65-*hrf*Δ parasites were challenged with 10^5^ WT iRBCs 4 weeks after the elimination of the mutant parasite from the blood (Fig. 2). A control group of mice that was not pre-infected nor treated by antimalarial drugs was inoculated at the same time with 10^5^ WT iRBCs. As obtained with WT parasite challenges in protected mice without drug treatment, infection with *Pb*NK65-*hrf*Δ parasites allowed the development of a long-lasting immune protection despite a prior drug treatment. This result shows that a primary infection with WT parasites followed by a drug cure does not hamper the efficacy of *Pb*NK65-*hrf*Δ-induced protection.

### Pattern of cytokines associated with *Pb*NK65*-hrf*Δ induced protection

**Figure 3:**
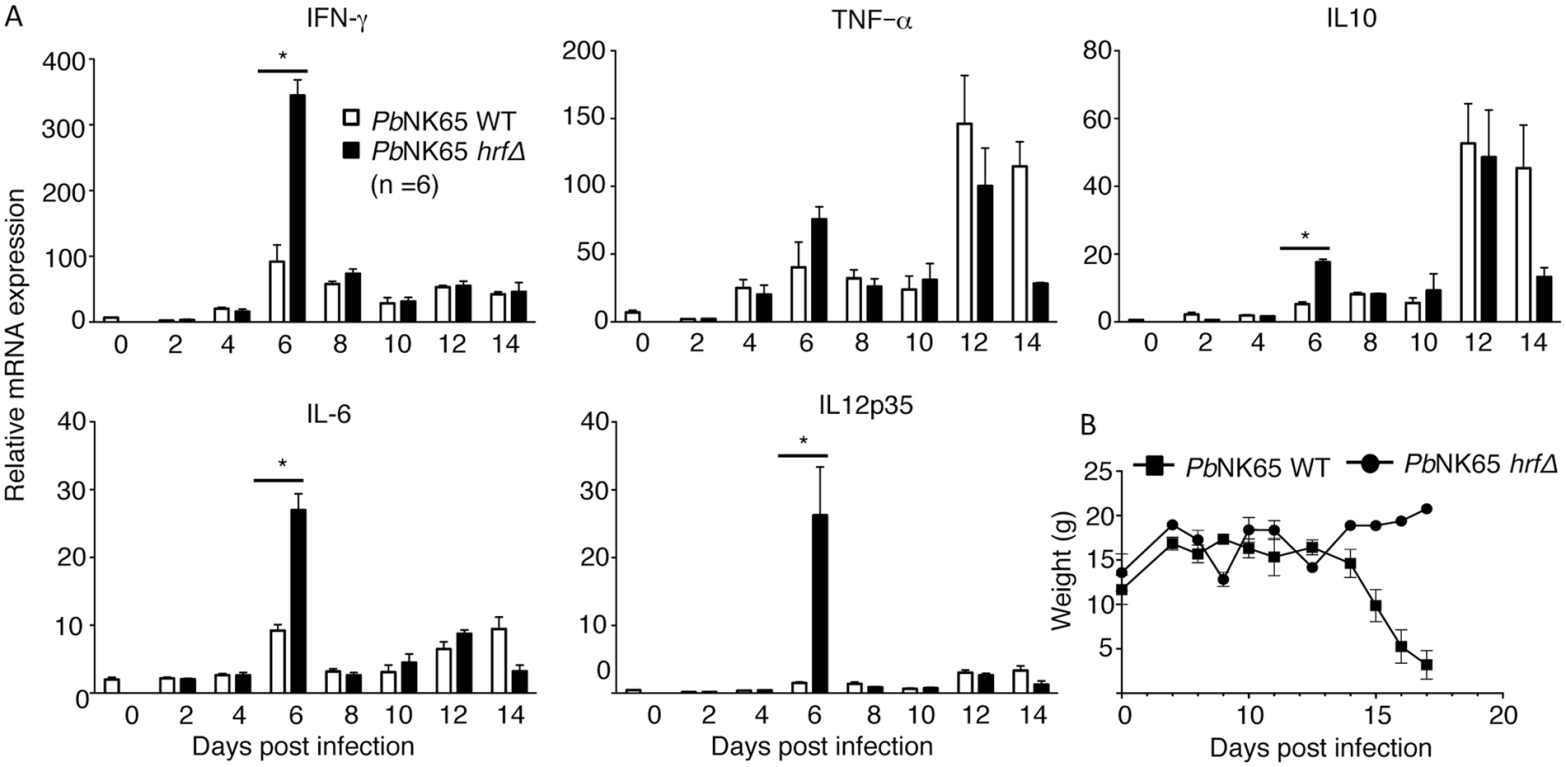
Immune response genes are differentially regulated by WT and *hrf*Δ parasites. (**A**) mRNA levels (RT-qPCR) normalized to HPRT of cytokine production in spleen cells measured at different time points p.i., every other day from day 2 to day 14 p.i., from mice infected with 10^5^ WT or *Pb*NK65 *hrf*Δ iRBCs. (**B**) Determination of body weight measured over time during C57BL/6 mice infection. Error bars, SEM. Data are representative of two independent experiments with 5 mice per group. (*p < 0.03; Mann Whitney test).

It is known from previous studies that Th1 cytokines IL-12, IFN-γ, and TNF-α were shown to confer immunity against blood-stage *Plasmodium* infection^35^. To examine whether the infection of C57BL/6 mice with either the WT or *Pb*NK65 *hrf*Δ parasite induces a particular set of cytokines, we first examined the mRNA expression of a variety of cytokines by RT-PCR in the spleen of infected mice at various time intervals after infection, starting from day 2 until day 14, p.i. (Fig. 3A). A higher expression of IFN-γ, IL-10, IL-6 and IL-12p35 cytokines was noticed at day-6 p.i. in mice infected with *Pb*NK65 *hrf*Δ as compared to WT parasites (Fig. 3A). A more detailed analysis of this group of cytokines was performed at day 6 p.i. in the liver and in the spleen of infected mice. Results showed that IL-23, EBI-3 (IL-27 beta subunit), IL-12p40, IL-12p35, IFN-γ, IL-6, and IL-10 mRNA expression were all increased both in the liver and in the spleen during *Pb*NK65 *hrf*Δ infection as compared to WT infection (Supplementary Fig. 2A,B). At the protein level, higher levels of IFN-γ, IL-12p70, and IL-6, as measured by ELISA at day 6 p.i., were confirmed in the plasma of *Pb*NK65 *hrf*Δ infected mice as compared to WT parasite-infected mice (Supplementary Fig. 2C). This burst of pro-inflammatory cytokines induced by *Pb*NK65 *hrf*Δ parasites was associated with a significant loss of weight of mice at day 8 p.i. (Fig. 3B), followed by the decrease in parasitemia and the weight normalization a few days later (day 10 p.i.). The loss of weight from day 4 to day 8 represented 10% of the weight of control mice at the same age. In contrast, mice infected with WT parasites did not show any loss of weight until day 8 (Fig. 3B). They displayed a delayed peak of TNF-α, IL-10 and IL-6 at day 12–14 p.i. and a drastic loss of body weight starting at day 14 (Fig. 3B).

### Protection conferred by mutant parasites is dependent on effector CD4^+^ T cells

**Figure 4:**
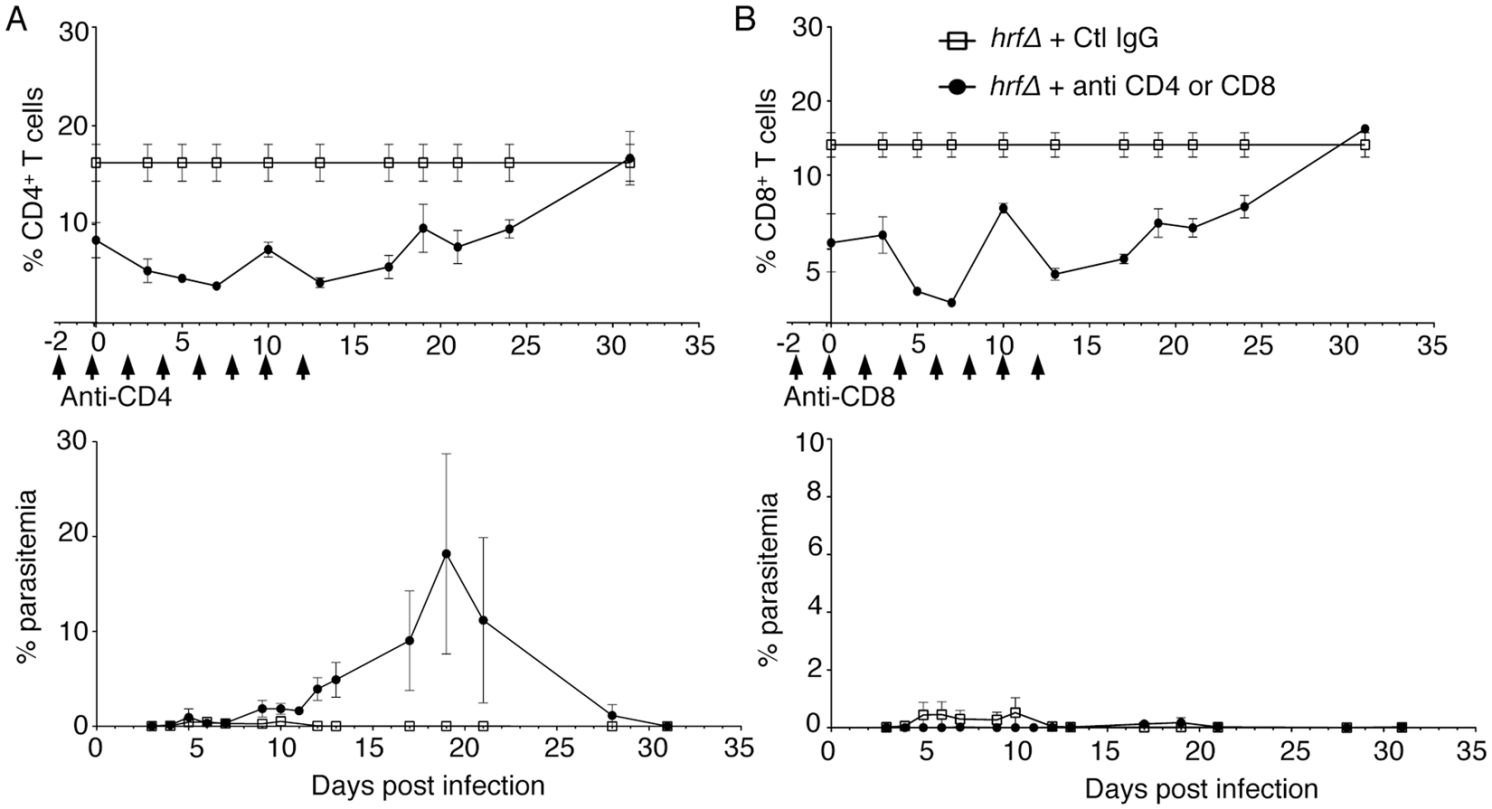
Influence of CD4^+^ or CD8^+^ T cells depletion on parasite development in protected mice. *Pb*NK65 *hrf*Δ iRBCs-protected mice were treated either with IgG or with anti-CD4− (**A**) or with anti-CD8-depleting Abs (**B**) 2 days prior to a challenge with 10^5^ iRBCs WT parasites followed by 7 injections of IgG, anti-CD4 or anti-CD8 Abs administered every other day after the infection. Anti-CD4 and CD8 treatment was discontinued at day 12 p.i. Parasitemia as well as determination of cell counts in the blood were recorded over time. Error bars, SEM. Data are representative of two independent experiments with 5 mice per group.

We have previously shown the critical role of T cells in the development of an anamnestic response in mice previously infected with *Pb*NK65 *hrf*Δ1 parasites. Indeed, when CD3^+^ T cells were depleted, immunized mice were not protected^25^. In order to address whether the protection induced by *Pb*NK65 *hrf*Δ parasites was dependent on effector CD8^+^ and/or CD4^+^ T cells, protected mice were treated with normal mouse IgG, anti-CD8 or anti-CD4 depleting antibodies. Efficacy of CD4 and CD8 depletion was continuously monitored during administration of T-cell depleting antibodies and after this treatment was discontinued (Supplementary Fig. 3). Mice were then challenged with 10^5^ RBCs infected with WT *Pb*NK65 parasites (Fig. 4) and parasite growth and cell depletion efficacy were monitored daily by flow cytometry in blood samples. Interestingly, the measurement of parasitemia indicated a loss of parasite control upon treatment of protected mice with anti-CD4 antibody (Fig. 4A), but not with anti-CD8 antibody (Fig. 4B). WT parasite-challenged mice treated with control IgG remained parasite free. This suggests that the activation of CD4^+^ T effector lymphocytes, but not of CD8^+^ cells, is indispensable for protection. These results are in agreement with previous studies in animal models using depletion or adoptive transfer of different T cell populations, which highlighted the important role of CD4^+^ T cells in the development of protective immunity against blood stages^36, 37^.

### Protection induced by *Pb*NK65-*hrf*Δ parasites is associated with fewer T cells expressing PD-1

**Figure 5:**
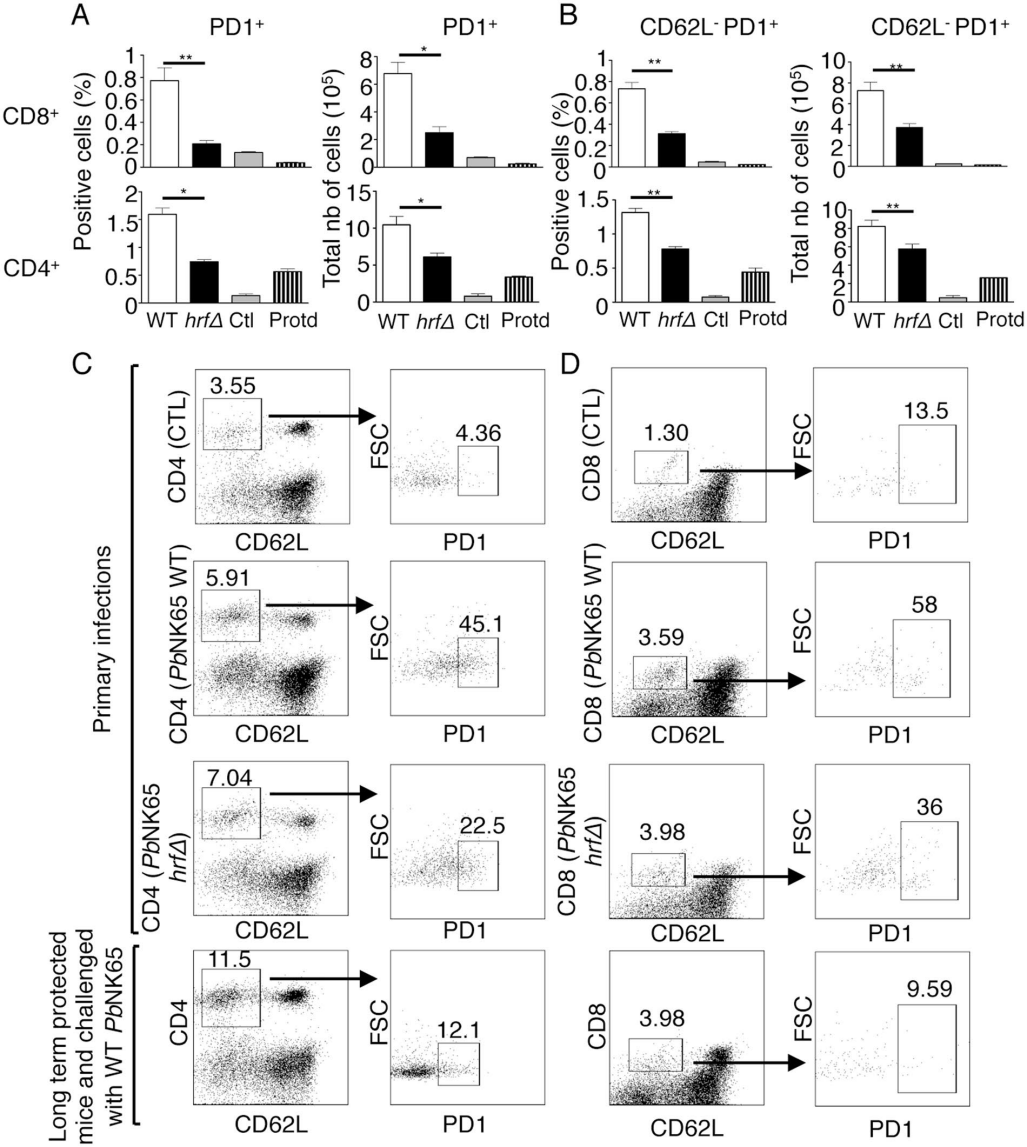
Protection induced by *Pb*NK65 *hrf*Δ parasites is associated with reduced induction of PD-1^+^CD8^+^CD62L^−^ and PD-1^+^CD4^+^CD62L^−^ cells in spleen. Naïve C57BL/6 mice were infected with 10^5^ iRBCs *Pb*NK65 WT or *hrfΔ* parasites and splenocytes were harvested either at day 6 p.i. or after a long-term protection (hatched bars, 8 months after initial infection with mutant parasites followed by challenges with WT parasites) Splenic leukocytes were stained with antibodies to CD4, CD8, PD-1 and CD62L and analysed by flow cytometry. Representative frequency and absolute number of splenic leukocytes showing expression of PD-1 on CD8^+^ and CD4^+^ T cells (**A**) which were further analysed for CD62L expression (**B**). (**C**,**D**) Gating strategy for FACS analysis of CD4^+^ or CD8^+^ gated splenoctyes, which were first assessed for the expression of CD62L. Then, the CD4^+^ CD62L^−^ and the CD8^+^ CD62L^−^ cells were analysed for PD-1 expression. Data are representative of two independent experiments with 5 mice per group. Error bars, SEM. (*0.0012 < p < 0.0095, **0.0106 < p < 0.0357; Mann Whitney test).

As previously observed^25^, mice lacking T cells were unable to control the parasitemia of *Pb*NK65 *hrf*Δ parasites suggesting that the activation of T lymphocytes may be indispensable for immune memory against malaria infection. To better characterize the molecular signatures of the T cell response and given that chronic malaria infection results in an increased frequency of T cells expressing surface markers of exhaustion such as programmed cell death-1 (PD-1)^12^, we asked whether the self-resolving nature of *Pb*NK65 *hrf*Δ infection could be correlated to a change in PD-1 expression on the surface of effector T cells. Analysis of PD-1 expression by flow cytometry at day 6 and at a later time point (8 months after the initial infection) (Fig. 5A) indicated that WT parasites induced a high proportion of PD-1^+^CD4^+^ and PD-1^+^CD8^+^ T cells in the spleen presumably representing a pool of T cells devoid of anti-parasite effector function. In contrast, *Pb*NK65 *hrf*Δ parasites induced two-fold and four-fold fewer PD-1^+^ CD4^+^ and PD-1^+^ CD8^+^ T cells, respectively at day 6 p.i. than WT parasites. A similar analysis was performed after a long-term protection in mice taken 8 months after the initial infection with *Pb*NK65 *hrf*Δ parasites and which received multiple challenges of the WT parasite. While PD-1 expression on CD4^+^ T cells remained at levels equivalent to that of KO parasite-infected mice at day 6 p.i, the expression level on CD8^+^ T cells was hardly detectable (Fig. 5A, Protd hatched bars). We further correlated PD-1 expression to the activation status of T cells using the cell surface marker CD62L, as low or undetectable levels of surface CD62L are indicative of T cell activation and effector function^38^. At day 6 p.i. we observed that in WT parasite-infected mice, activated CD4^+^CD62L^-^ and CD8^+^CD62L^−^ T cells expressing PD-1 represent about the double that of mice infected with *Pb*NK65 *hrf*Δ parasites (Fig. 5B). At late time points, 8 months after the initial infection, the number of PD-1^+^ activated CD8^+^ further decreased in *Pb*NK65 *hrf*Δ-infected mice, returning to the basal levels found in uninfected mice, whereas a residual number of PD-1^+^ activated CD4^+^ T cells persisted but at a significantly lower level as compared to early time points (day 6 p.i) (Fig. 5B, hatched bars). Detailed FACS plot and gating strategy is shown in Fig. 5C,D.

We further analysed PD-1 expression on “antigen-experienced“ CD4 and CD8 T cells based on the co-expression of CD11a^hi^ and CD49d^hi^ markers^39^ at day 6 post-infection with WT and mutant parasites. It is now well established that antigen-activated CD4 T cells downregulate CD62L and upregulate cell surface expression of integrins such as CD49d and CD11a allowing for their egress from lymph nodes and migration to the site of infection^40, 41^. We used this approach to examine to which extent antigen-experienced activated T cells expressed PD-1. While the percentage and total CD8^+^ CD11a^hi^ CD49d^hi^ cells were similar between WT- and mutant-infected mice, the proportion and the amount of cells expressing PD-1 were much lower in mutant- as compared to WT-infected mice (Supplementary Fig. 4A and B). In contrast, the percentage and total CD4^+^ CD11a^hi^ CD49d^hi^ cells were significantly higher in mutant- as compared to WT-infected mice, and the population of cells expressing PD-1 was also much more reduced in mutant-infected mice (Supplementary Fig. 4C and D). FACS plots and gating strategy for this analysis is shown in Supplementary Fig. 4E and F. To investigate whether r*Pb*HRF can directly modulate the expression of PD-1 on the surface of T cells, naïve mice were injected with 100 μg of r*Pb*HRF or BSA as a negative control and 48 h later, PD-1 surface expression was examined. We observed an upregulation of PD-1 on CD4^+^ and CD8^+^ T cell surface (Supplementary Fig. 5A) and on activated CD4^+^ CD62L^−^ and CD8^+^ CD62L^−^ T cells (Supplementary Fig. 5B) induced by the r*Pb*HRF. Taken together, these data demonstrate that infection with *Pb*NK65 parasites expressing *Pb*HRF is associated with an increased proportion of PD-1^+^ T cells in mice, indicative of T cell unresponsiveness.

### Infection with *Pb*NK65 *hrf*Δ parasites is associated with a marked increase in the number of effector memory B cells

**Figure 6:**
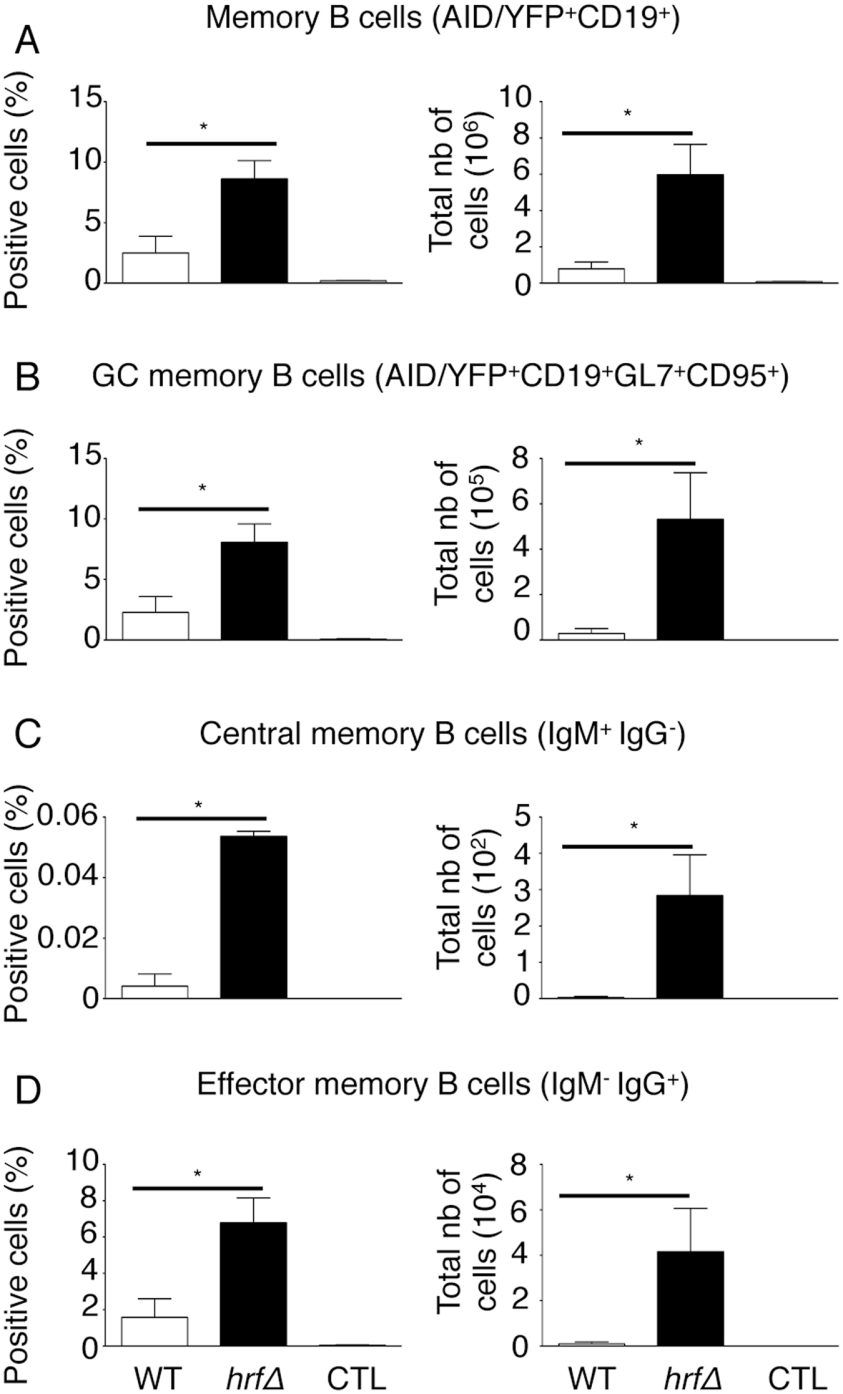
Frequency of memory B cells in *Pb*NK65-*hrf*Δ protected mice. Splenic B cells from naïve mice or 15 days p.i. with either WT or *hrf*Δ iRBCs were analysed fror their memory phenotype. Representative frequency and absolute number of AID/YFP^+^CD19^+^ (**A**), GC AID/YFP^+^CD19^+^ GL7^+^CD95^+^ (**B**) memory B cells. Representative frequency and absolute number, among GC B memory cell population, of IgM^+^IgG^-^ central memory B cells (**C**) or IgM^−^IgG^+^ effector memory B cells (**D**). Error bars, SEM. Data are representative of two independent experiments with 4 mice per group. (*p < 0.05; Mann Whitney test).

In a previous report^25^, in contrast to mice infected with the WT parasite, we found that mice infected with the mutant parasite developed a strong and long lasting antibody response associated with the opsonisation and the priming of CD11b^+^ cells that were essential for infection resolution^25^. These antibodies recognized multiple *P. berghei* antigens in contrast to sera from WT *Pb*NK65–infected mice or from naive mice^25^. Protection from reinfection relies on the establishment of an efficient secondary immune response that requires the generation and selection of “memory” B cells originated from T cell-dependent B cell response through two molecular mechanisms: immunoglobulin isotype recombination and somatic hyper mutations, both dependent on the expression of the GC-specific enzyme activation-induced cytidine deaminase (AID) in the germinal centres (GCs). In order to investigate the generation of the immunological memory during *Pb*NK65 *hrf*Δ infections, we analyzed further the memory B cell generation. We used a strain of mice transgenic for a construct enabling permanent marking of AID activation through YFP expression, and consequently tracking of long-term memory B cells containing populations of “central” memory (AID/YFP^+^CD19^+^IgM^+^IgG^−^) and “effector” memory (AID/YFP^+^CD19^+^IgM^−^IgG^+^) B cells^42, 43^. AID/YFP mice were infected i.p with 10^5^ of either WT or *Pb*NK65 *hrf*Δ iRBCs. At day 20 post-infection mice, once *Pb*NK65 *hrf*Δ parasite was eliminated and memory B cells were generated, splenic B cell populations were analysed by flow cytometry using a combination of cell surface markers according to a gating strategy on CD19^+^ AID-YFP^+^ cells followed by gating on GL7^+^ CD95^+^ cell population and finally gating on IgG^+^ or IgM^+^ cell populations (Supplementary Fig. 6). As shown in Fig. 6, significantly higher total memory and GC memory B cells, central and effector memory B cells were observed in mice infected with the mutant parasite as compared to the WT parasite (Fig. 6A,B,C, and D, respectively). These results reinforce the hypothesis that B cells are important effectors in the immune response developed in C57BL/6 mice against *Pb*NK65 *hrf*Δ parasites which act in tandem with CD4^+^ T cells.

## Discussion

In recent years, in addition to the development of subunit vaccines and radiation-attenuated sporozoites (RAS), researchers have used rodent models to test the efficacy of GAPs as vaccines against pre-erythrocytic and blood-stage malaria infections. Most studies have focused on pre-erythrocytic GAPs, which include parasite mutants blocked early during their development in the liver, such as those lacking integrity of the parasitophorous vacuole, or late during development, such as those with a deficient fatty acid biosynthesis type II pathway^44^. Some of these pre-erythrocytic GAPs are currently being tested in humans, e.g., the early-blocked *P. falciparum* Δ*p52*Δ*p36*Δ*sap1* ^45^. In comparison, fewer GAPs have been constructed and analysed as blood-stage vaccines.

We have investigated the protection mediated by an erythrocytic GAP depleted of the gene encoding the immunomodulatory and secreted molecule HRF, using the parasite strain *P. berghei* NK65 that does not cause cerebral malaria and rapid death. In earlier work, we found that blood-stage infection by the mutant self-resolved at day 12 p.i., displaying an immune signature that comprised elevated IL-6 levels, activation of T and B cells, and antigen-specific IgG2c production^25^. Importantly, the *Pb*NK65*hrf*Δ GAP was found to induce strong, long-lasting, cross-stage and cross-species protection against subsequent malaria infections, suggesting that the immune effector mechanisms were directed against antigens shared by different stages and parasite species. In the present work, we showed that the protection induced by the mutant was dependent on CD4^+^, but not CD8^+^ T cells, and was associated with reduced numbers of PD-1^+^ T cells and higher numbers of memory B cells.

The reasons for the abortive infection and subsequent protection by *Pb*NK65 *hrf*Δ parasites are likely multifactorial. Nevertheless, induction of IL-6 upon infection with the *Pb*NK65 *hrf*Δ parasite appears to be a key mechanism that is encountered at both pre-erythrocytic and erythrocytic stages and regardless of the parasite genetic background, i.e. *Pb*NK65 *hrf*Δ or *Pb*ANKA *hrf*Δ^25, 26^. Indeed, similar to infection with *Pb*ANKA *hrf*Δ sporozoites^34^, a delayed development of *Pb*NK65 *hrf*Δ sporozoites in the liver was associated with a sharp peak of IL-6 up-regulation in this tissue at 48 h p.i (Fig. 1D). This indicates that up-regulation of IL-6 following infection with mutant parasites regardless of the parasite strain is a hallmark of the HRF gene product, which was shown to directly control IL-6 production^25^. This later property is key to understand the protective mechanisms elicited by *Pb*NK65 *hrf*Δ parasites. Indeed, concomitantly with, and dependent on IL-6 production, we detected a higher production of IL-12 cytokine family including IL-12p35, IL-12p40, IL-23, and Ebi3. During malaria infection, early non-specific immune responses can be augmented by the release of IL-12 from splenic macrophages^46, 47^, and the activation of these macrophages by the production of IFN-γ results in an increased phagocytic activity and the killing of malaria parasites. A parallel can be made in the human situation, since during *P. falciparum* infection children with mild malaria infection have higher levels of plasma IL-12 when compared to children with severe malaria infection, and the levels of IL-12 are inversely correlated with parasitemia and the numbers of malaria pigment-containing neutrophils^48, 49^. A prominent role of IL-6 in the induction of Th1 cell response has been recently documented *in vivo*, which enables T cell activation by making CD4^+^ T cells less sensitive to the suppressive activity of Tregs, promotes the generation of functional memory CD4^+^ T cells, and provides help to B cells^50^.

Two additional key findings were presented in this work: first, a significant proportion of CD4^+^ and CD8^+^ T cells have their PD-1 expression upregulated during infection with WT parasites, in contrast to infection caused by *hrf*Δ parasites. *Pb*NK65 *hrf*Δ infections correlated with a reduction of CD4^+^PD-1^+^CD62L^−^ and CD8^+^PD-1^+^CD62L^-^ cells, which are memory effector T cells. The PD-1 marker was found early during infection, as early as day 6, and lasted throughout infection, suggesting a possible influence of lack of PD-1 on both clearance of primary infection and induction of lasting protection. Signalling through the PD-1 receptor is thought to “exhaust” CD4^+^ and CD8^+^ T cells, leading to poor effector functions and expression of inhibitory receptors^51–53^. Interestingly, an involvement of PD-1 in the control of malaria blood-stage infection has already been reported: in *P. falciparum* infections, higher expression of PD-1 was associated with T cell dysfunction, and therapeutic blockade of PD-1 ligand in a murine model of infection rapidly cleared blood-stage malaria in a B- and T-cell dependent manner^12^. In the *P. chabaudii* rodent model of chronic blood-stage infection, parasite-specific CD8^+^ T cells undergo significant PD-1-dependent exhaustion (up to 95% reduction), which exacerbates acute blood-stage infection and drives chronic disease^54, 55^. Although a plethora of reports indicate that PD-1/PD-L1 pathway regulates immune suppression by inducing apoptosis of activated T cells or facilitate T cell anergy and exhaustion, it remains that the primary function of PD-1 is to block the downstream signalling events triggered by antigen/MHC engagement of the TCR resulting in impaired T cell activation and IL-2 production. The low frequency of CD4 and CD8 T cells expressing PD-1 in mice that were infected with the *Pb*NK65 *hrf*Δ parasite is consistent with the propensity of a higher cytokine production in these mice. This is also consistent with the long-lasting T cell memory observed in the protected mice, which is supported by the fact that signaling through PD-1 was shown to prevent the conversion of functional T effector memory cells into central memory cells^56^ and, thus, reduces long-term immune memory that might protect against future wild-type parasite challenges. Interestingly, the fact that the upregulation of PD-1 expression on CD4 and CD8 T cells was not only the result of a subtractive effect between the mutant parasite and the wild-type parasite, but was recapitulated by using the recombinant protein HRF, suggests that HRF may act directly on CD4^+^ and CD8^+^ T cells (Supplementary Fig. 5A,B). Because HRF also down-regulates IL-6 production^25^, a relationship may exist between IL-6 and PD-1 expression. A parallel can be made with the recent finding that IL-6 modulates CD4^+^ T cell reactivity to PD-L1 by inducing the release of a soluble form of PD-1^57^. The apparent reduced number of T cells expressing PD-1 in mice infected with *Pb*NK65 *hrf*Δ parasites could therefore be explained by a shedding of PD-1 from the T cell surface.

The second important finding in this work is that anti-parasite antibodies could not be detected at any time of WT infection, during which the B cell compartment seems to be completely non-functional. Recently, it was demonstrated that severe blood infection with *Pb*ANKA strain inhibited T helper cell differentiation and germinal center formation^58^. In agreement with this, the aberrant B cell memory and the lack of maintenance of specific antibody response upon infection with WT *Pb*NK65 parasite were completely reverted in mice infected with the *Pb*NK65 *hrf*Δ parasite. It is generally recognized that a response to a T cell-dependent antigen results in B cell memory taking place in germinal centers. At this particular site, affinity maturation and class switching of antibody receptors are initiated by the germinal center-specific enzyme called activation-induced cytidine deaminase (AID)^59^. Using AID/YFP reporter mice, we could determine the fate of germinal center memory B cells (AID/YFP^+^ CD19^+^ GL7^+^ CD95^+^) in mice infected with WT *Pb*NK65 as compared with *Pb*NK65 *hrf*Δ parasites. Consistent with previously obtained data, a significantly higher proportion and number of memory B cells were observed in mice infected with mutant parasites. A thorough analysis of memory B cell sub-populations revealed a higher proportion and number of both central (IgM^+^ IgG^−^) and effector (IgM^−^ IgG^+^) memory B cells in mice protected by infection with *Pb*NK65 *hrf*Δ parasites. The role of CD4^+^ and CD8^+^ T-cell and antibody responses, particularly against blood-stage infection, remains elusive mostly due to the diversity of experimental protocols, the biology of the *Plasmodium* strains used, and host genetics. As an example, the variety of protective immune mechanisms is reflected by the predominance of T cells in *P. chabaudi* infections^60^ and of antibodies in *P. yoelii* infections^61^. Among T cells, CD4^+^ T cells are known to modulate the function of several effector cells including CD8^+^ T cells and macrophages and help B cells to produce antibodies, altogether participating to the generation of protective responses against *Plasmodium* infection. In the present work, it was striking to observe that mostly CD4^+^ T cells, but not CD8^+^ T cells are key players in the acquisition of protective immunity induced by *Pb*NK65 *hrf*Δ parasites. Similarly, vaccination with chemically attenuated *P. yoelii* 17X demonstrated the crucial role of CD4^+^ T cells after blood-stage parasite challenge, with the depletion of CD4^+^ T cells, but not of CD8^+^ T cells resulting in a loss of protection^62^. The same group demonstrated earlier that CD4^+^ T cell-depleted mice previously vaccinated with *P. chabaudi* iRBCs attenuated with centanamycin all succumbed, whereas no change in their level of immunity was observed when CD8^+^ T cells were depleted from immune mice^63^. It remains unclear why CD4^+^ but not CD8^+^ T cells are selectively associated with protection, although equally fewer CD4^+^ and CD8^+^ T cells expressing PD-1 were associated with *Pb*NK65 *hrf*Δ infection (Fig. 5A,B). Nevertheless, in our system protective mechanisms are dominated by two concerted effector mechanisms: effector CD4^+^ T cells and antibody-producing B cells that ultimately promote CD11b phagocytic activity.

Our work, the present data and our previous reports^25, 34^, explored in detail all compartments of innate and adaptive immune responses associated with protection elicited by the *Pb*NK65 *hrf*Δ parasite. Since such detailed analysis of effector immune mechanisms are not available for blood-stage GAP reported by other groups, it cannot be conclusively established whether or not common protective mechanisms are induced by all self-resolving GAP infections. This question remains open until a systematic analysis of various mutants can be performed side by side in one single experimental setting. Furthermore, it can be anticipated that therapeutic or vaccine interventions in naïve individuals and in individuals with an infection history may implicate distinct physiological states of the host immune system, and therefore imply different outcomes in terms of resistance or susceptibility to the pathogen and response to vaccination. In the present work, the vaccine efficacy of the *Pb*NK65 *hrf*Δ parasite infection, which was initially demonstrated in naïve mice with no history of infection, was found to persist in mice infected with the WT parasite and cured by drug treatment prior to *Pb*NK65 *hrf*Δ vaccination. This would suggest that this blood-stage vaccination approach may be efficient even in individuals that have already been infected in natural conditions and who repeatedly received antimalarial drugs.

## Methods and Materials

### Ethics statements

All animal care and experiments described in the present study involving mice were conducted at the Institut Pasteur, approved by the ‘Direction Départementale des Services Vétérinaires’ de Paris, France (Permit Number N° 75–066 issued on September 14, 2009) and performed in compliance with institutional guidelines and European regulations (http://ec.europa.eu/environment/chemical?s/lab_animals/home_en.htm). A statement of compliance with the French Government’s ethical and animal experiment regulations was issued by the Ministère de l’Enseignement Supérieur et de la Recherche under the number 00218.01.

### Rodents

Five- to eight-week-old wild-type female C57BL/6 J Rj and Swiss Webster (SW) mice were purchased from Janvier laboratory (Le Genest-Saint-Isle, France). Transgenic AID/YFP^42^ were kindly provided by Dr. Antonio A. Freitas (Institut Pasteur, Paris, France).

### Parasites

Mice were inoculated with red blood cells infected (iRBCs) or sporozoites collected from salivary glands of infected *Anopheles stephensi* with either GFP-transgenic *Plasmodium berghei* (*Pb*) NK65 wild-type or mutant (*hrf*Δ) GFP-transgenic clones.

### Mouse infections

Mice were infected with blood stages of either GFP-transgenic *P. berghei* NK65 or *Pb*NK65 *hrf*Δ parasites by injecting 10^5^ infected red blood cells (iRBCs) intraperitoneally (i.p) or 10^3^ sporozoites intravenous (i.v.). After the infection, blood samples were taken daily from the tail and the parasitemia was assessed by flow cytometry and the results expressed in percentage of iRBC. Infected mice were monitored for clinical symptoms of the disease: weight loss, anemia, fever and death.

### Drug treatment

Once mice infected with *Pb*NK65 WT iRBCs reach 2% of parasitemia were treated with for three consecutive days with 6 mg/kg of WR99210 (Sigma-Aldrich, Saint Louis, USA) by subcutaneous (s.c.) injections. Once the parasites were completely eliminated from blood stream mice were infected either with 10^5^ *Pb*NK65 WT or *hrf*Δ iRBCs. The group of mice who received the *Pb*NK65 *hrf*Δ and eliminated it from the blood stream were additionally challenged with 10^5^ *Pb*NK65 WT iRBCs.

### Preparation of total RNA and reverse transcription-quantitative PCR (RT-qPCR) analysis of mRNA

The livers and spleens of C57BL/6 J mice infected with WT or *Pb*NK65 *hrf*Δ1 parasites were surgically removed 48 h, 72 h, 96 h and 120 h p.i. or at day 2, 4, 6, 8, 10, 12, 14 and 20 p.i., respectively. Total RNAs were extracted from the spleen as well from the liver samples using the guanidinium-thiocyanate-phenol-chloroform method (all Invitrogen, Waltham, MA, USA). RNA was thereafter reverse transcibed by PCR (temparature profile: 65 °C for 5 min, 42 °C for 50 min, 70 °C for 15 min) using 100U SuperScript™ II reverse transcriptase (RT) (Invitrogen, Waltham, MA, USA), 40U RNAse Inhibitor and 2 μM oligo(dT) 18 S rRNA primer (Eurofins MWG Operon, Ebersberg, Germany) per sample. The expression levels of diverse transcripts were analyzed by real time RT-qPCR using Power SYBR® Green PCR Master Mix (Applied Biosystems Foster City, CA, USA) and various primers sets (Table S1). All reactions were performed in the ABI PRISM 7000 Sequence Dectection System Real Time PCR machine (temparature profile: 50 °C for 2 min, 95 °C for 10 min, 40 cycles of 95 °C for 15 s and 60 °C for 1 min). The relative abundance of parasite and cytokines rRNA in the spleen was calculated using the ∆C_t_ method, and expressed as 2^−∆Ct^. The mouse hypoxanthine phosphoribosyltransferase (HPRT) gene was used as an internal control for the variation in input RNA amounts. No template control (NTC) was included to ensure that there was no cross-contamination during sample preparation.

### Detection of specific antibodies, cytokines, and chemokines in the serum of infected mice

To detect parasite-specific antibodies, 96-well plates (Nunc-immuno plate; Thermo Scientific, Rockford, IL) were coated with parasite protein extract from asexual blood stages in carbonate buffer, pH 9.6, for 2 h at 37 °C. After the plates were saturated with 1% (w/v) pork gelatine, each serum was assayed at serial dilutions and incubated overnight for 2 h at 37 °C. Specific binding was detected using HRP-conjugated goat anti-mouse secondary antibody (Cell Signalling technology®, Danvers, MA) followed by the addition of o-phenylenediamine dihydrochloride (OPD) substrate (Sigma-Aldrich; St.Louis, MO). Hydrogen chloride (HCl) 1 N was used to block the reaction. The optical density (OD) was read at 490–655 nm. Each sample was tested against non-immune serum and PBS as background controls. Amounts of IL-12p70, IFN-γ and IL-6 in the serum were analysed by cytokine-specific ELISA kits (BD Biosciences, Mountain View, CA).

### Flow cytometry analysis of spleen leukocytes

Spleens were mechanically disrupted in 2 ml PBS and cells were filtered through a 70-mm strainer (BD Falcon). Erythrocytes on the cell suspension were lysed using Gey’s solution for 5 min of incubation on ice and after washed two times in PBS. Single-cell suspension were stained for FACS analysis according to standard protocols in cold PBS containing 2% FCS and 0.01% sodium azide (FACS buffer) with the following monoclonal antibodies conjugated to fluorescein isothiocyanate (FITC), phycoerythrin (PE), phycoerythrin-cyanine 5 (PeCy5), phycoerythrin-cyanine 7 (PeCy7), peridinin chlorophyll protein-cyanine 5.5 (PerCp-cy5.5), PerCp-eFluor 710, allophycocyanine (APC), Alexa Fluor 700 (AF700) and Qdot-605: anti-CD4 (FITC), anti-CD4 (AF700), anti-CD11a (FITC), anti-CD8a (PE), anti-GL7 (PE), anti-CD49d (PerCp-eFluor 710), anti-CD62L (PeCy5), anti-CD95 (PeCy7), anti-IgM (PerCp-cy5.5), anti-PD-1 (APC), anti-IgG (APC) and CD19 (Qdot-605) (all antibodies from BD Bioscience, Mountain View, CA). Before staining, a total of 5 × 10^5^ living cells, were treated with Fc-Block (clone 2.4 G2, BD Bioscience, Mountain View, CA). Dead cells were excluded during analysis according to their light-scattering characteristics. Data and analyses were performed with LSRFortessa (Becton Dickinson, Grenoble, France) or four-color FACSCalibur (Becton Dickinson, Grenoble, France) using FlowJo software (Tree Star, Ashland, OR, USA) or CellQuest Pro software (Becton Dickinson, Grenoble, France).

### *In vivo* cell depletion

To determine if the protection induced by *Pb*NK65 *hrf*Δ is dependent on effector CD4^+^ or CD8^+^ T cells, cell-specific depletion experiments were performed. C57BL/6 J Rj protected mice were injected i.p. with 20 μg of anti-CD8 clone 53–6.7 Armenian hamster IgG (eBioscience, San Diego, CA) or 100 μg of rat anti mouse CD4 clone GK1.5 (ATCC® TIB207™) 48 h before the infection with *Pb*NK65 WT followed by 6 injections administered every other day after the infection. The cell depletion was followed and confirmed every day by taking 10 μl of blood from the tip of the mouse tail and analysed by flow cytometry.

### Statistical analysis

All data were analyzed using GraphPad Prism 5.0 software. Unparied data between two groups at a specific time point were analysed by Mann-Whitney test for nonparametric analysis when data did not fit a Gaussian distribution. A *p-*value of < 0.05 was considered to be statistically significant. All experiments were replicated several times as indicated in the figure legends.

## Electronic supplementary material

Supplementary information
