# Supplementary material for: Immunological memory to blood-stage malaria infection is controlled by the histamine releasing factor (HRF) of the parasite
Source: Sci Rep. 2017 Aug 22;7:9129. doi: 10.1038/s41598-017-09684-2 (PMC5567273; doi:10.1038/s41598-017-09684-2)

Immunological memory to blood-stage malaria infection is controlled by the histamine releasing factor (HRF) of the parasite

Claudia Demarta-Gatsi[1,2,3], Roger Peronet[1,2,3], Leanna Smith[1,2,3], Sabine Thiberge[4], Robert Ménard[4], Salaheddine Mécheri[1,2,3,5]

[1] Institut Pasteur, Unité de Biologie des Interactions Hôte Parasites,[2] CNRS ERL9195 and [3] INSERM U1201, Paris F-75015, France
[4] Institut Pasteur, Unité de Biologie et Génétique du Paludisme, F-75015 Paris, France

Short title: HRF mutant malaria parasite boosts immune memory

[5] Correspondence : Dr S. Mécheri, BIHP Unit, Institut Pasteur, 28 rue du Dr Roux, Paris 75724 Cedex 15.
Phone: 331 45 68 82 45 , Fax: 331 44 38 95 21, e-mail: smecheri@pasteur.fr

Supplementary Figure legends

Figure S1. Infection with HRF-deficient sporozoites ensures long-lasting cross-stage protection.

Mice taken from the experiment shown in figure 1B and protected upon inoculation with *Pb*NK65 *hrf*Δ iRBCs (A) or with sporozoites (B), were challenged with 10^5 WT *Pb*NK65 iRBCs at day 36 p.i. where control naïve mice were also infected with 10^5 WT *Pb*NK65 iRBCs as indicated by arrows at day 60 (A) and day 35 (B). Parasitemia was measured over time. Error bars, SEM. Data are representative of three independent experiments with 5 mice per group.

Figure S2. Cytokine production during WT and *Pb*NK65 *hrfΔ* blood-stage infection.

mRNA levels of IL-23, EBI-3, IL-12p35, IL-12p40, IFN-γ, IL-6 and IL-10 in the spleen (A) and in the liver (B) of naïve, or *Pb*NK65 WT and *Pb*NK65 *hrf*Δ infected mice at day 6 p.i. were determined by RT-qPCR analyses. The levels of each mRNA were normalized to HPRT. (C) Circulating levels of IL-12p70, IFN-γ and IL-6 in the serum of naïve mice or mice infected with *Pb*NK65 WT or *Pb*NK65 *hrf*Δ iRBCs were measured by ELISA at day 6 p.i. CTL: mRNA and sera from naive mice. Error bars, SEM. Data are representative of four independent experiments with 5 mice per group. (* $p < 0.01$; ** $p < 0.03$; Mann Whitney test)

Figure S3. Assessment of leukocyte depletion.

In vivo depletion of $CD4^+$ or $CD8^+$ T cells in protected mice using anti-CD4 or anti-CD8 antibody was assessed by measuring daily the percentage of residual (A) $CD4^+$ or (B) $CD8^+$ T cells in the blood by FACS analysis. Typical analysis performed at day 12 post treatment is shown in this figure. Two independent experiments with 5 mice per group.

Figure S4. $CD11a^{hi}CD49d^{hi}$ $CD4^+$ and $CD8^+$ T cells express PD-1 during infection with *Plasmodium* parasites.

(A, B) Representative frequency and absolute number of respectively $CD8^{+}CD11a^{hi}CD49d^{hi}$ and $CD8^{+}CD11a^{hi}CD49d^{hi}PD\text{-}1^{+}$ splenic leukocytes at day 6 p.i. with either 10^{5} WT or *hrfΔ* iRBCs. (C, D) Representative frequency and absolute number of respectively $CD4^{+}CD11a^{hi}CD49d^{hi}$ and $CD4^{+}CD11a^{hi}CD49d^{hi}PD\text{-}1^{+}$ splenic leukocytes at day 6 p.i. with either 10^{5} WT or *hrfΔ* iRBCs. Flow cytometric profiles of (E) $CD8^{+}$ and (F) $CD4^{+}$ T cells expressing CD11a and CD49d. The dot plots where used first to indicate the gating strategy used to assess the $CD11a^{lo}CD49d^{lo}$ (red gate) and $CD11a^{hi}CD49d^{hi}$ (black gate) T cells population and then the expression of PD-1 on $CD11a^{hi}CD49d^{hi}$ on T cells. Numbers in the plots indicate the proportions of gated cells. Error bars, SEM. Data are representative of two independent experiments with 5 mice per group. (* $p = 0.0286$, ** $p < 0.05$; Mann Whitney test).

Figure S5

(A) Representative frequency and absolute number of $CD8^{+}PD\text{-}1^{+}$ and $CD4^{+}PD\text{-}1^{+}$ splenic leukocytes 48h after recombinant *Pb*HRF administration (i.v.). (B) Representative frequency and absolute number of $CD8^{+}CD62L^{-}$ and $CD4^{+}CD62L^{-}$ splenic leukocytes expressing the PD-1 receptor 48h after recombinant *Pb*HRF administration (i.v.). Error bars, SEM. Data are representative of two independent experiments with 5 to 8 mice per group. (* $0.0006 < p < 0.0061$, ** $0.0121 < p < 0.0317$, *** $p < 0.05$; Mann Whitney test).

Figure S6. Flow cytometric analysis of IgG and IgM expression by AID/YFP^{+} GC memory B cells.

Flow cytometric analyses of $AID^{+}CD19^{+}$ splenic B cells from naive mice (upper panels) and mice infected with 10^{5} *Pb*NK65 WT (middle panels) or *Pb*NK65 *hrfΔ* (lower panels) iRBCs. Transgenic AID/YFP mice were first assessed for the expression of GL7 and CD95 positive cells. Then the $GL7^{+}$ $CD95^{+}$ cells were analysed for IgM and IgG expression. Numbers in the plots indicate the proportions of gated cells.Two independent experiments with 4 mice per group were performed.

Supplementary table 1: List of oligonucleotides used for RT-qPCR analyses.

Primer	Fw/Rev	Sequence
Pb 18S	Fw<br>Rev	ATTAATCTTGAACGAGGAATGGCT<br>TCAATCGGTAGGAGCGACG
Pb LSP2	Fw<br>Rev	GCCAAATGCTAAACCTAATG<br>TGGGTTTGTATTGTATGCAC
Pb HSP70	Fw<br>Rev	TGCAGCTAATCAAACTC<br>ACTTCAATTTGTGGAACACC
mu IL-23	Fw<br>Rev	CCACCAGGACTCAAGGACAACA<br>GCAGGCTCCCCTTTGAAGA
mu EBI3	Fw<br>Rev	CAGAGTGCAATGCCATGCTCC<br>GCCACACCGAGCCTGTAAGT
mu IL-12p35	Fw<br>Rev	TACTAGAGAGACTTCTTCCACAACAAGAG<br>GATTCTGAAGTGCTGCGTTGAT
mu IL-12p40	Fw<br>Rev	GGAAGCACGGCAGCAGAATA<br>AACTTGAGGGAGAAGTAGGAATGG
mu IFN-γ	Fw<br>Rev	AAAGGATGCATTCATGAGTATTGC<br>CGCTTCCTGAGGCTGGATT
mu TNF-α	Fw<br>Rev	CAT CTT CTC AAA ATT CGA GTG ACA A<br>TGG GAG TAG ACA AGG TAC AAC CC
mu IL-6	Fw<br>Rev	AAAGAAATGATGGATGCTACCAAAC<br>CTTGTTATCTTTTAAGTTGTTCTTCATGTACTC
mu IL-10	Fw<br>Rev	GGCGCTGTCATCGATTTCTC<br>GACACCTTGGTCTTGGAGCTTATTAA
mu HPRT	Fw<br>Rev	CTGGTGAAAAGGACCTCTCG<br>TGAAGTACTCATTATAGTCAAGGGCA

Supplementary figure 1

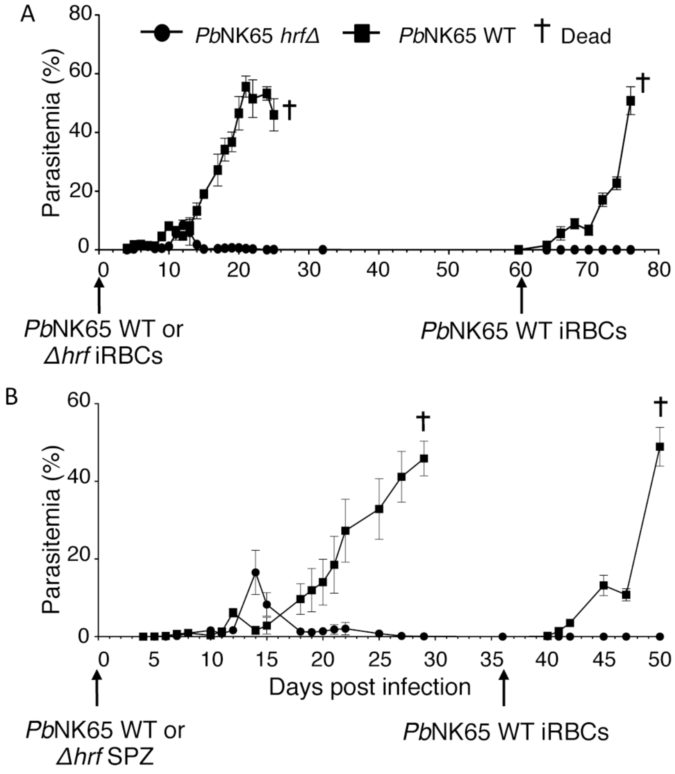

Supplementary figure 2

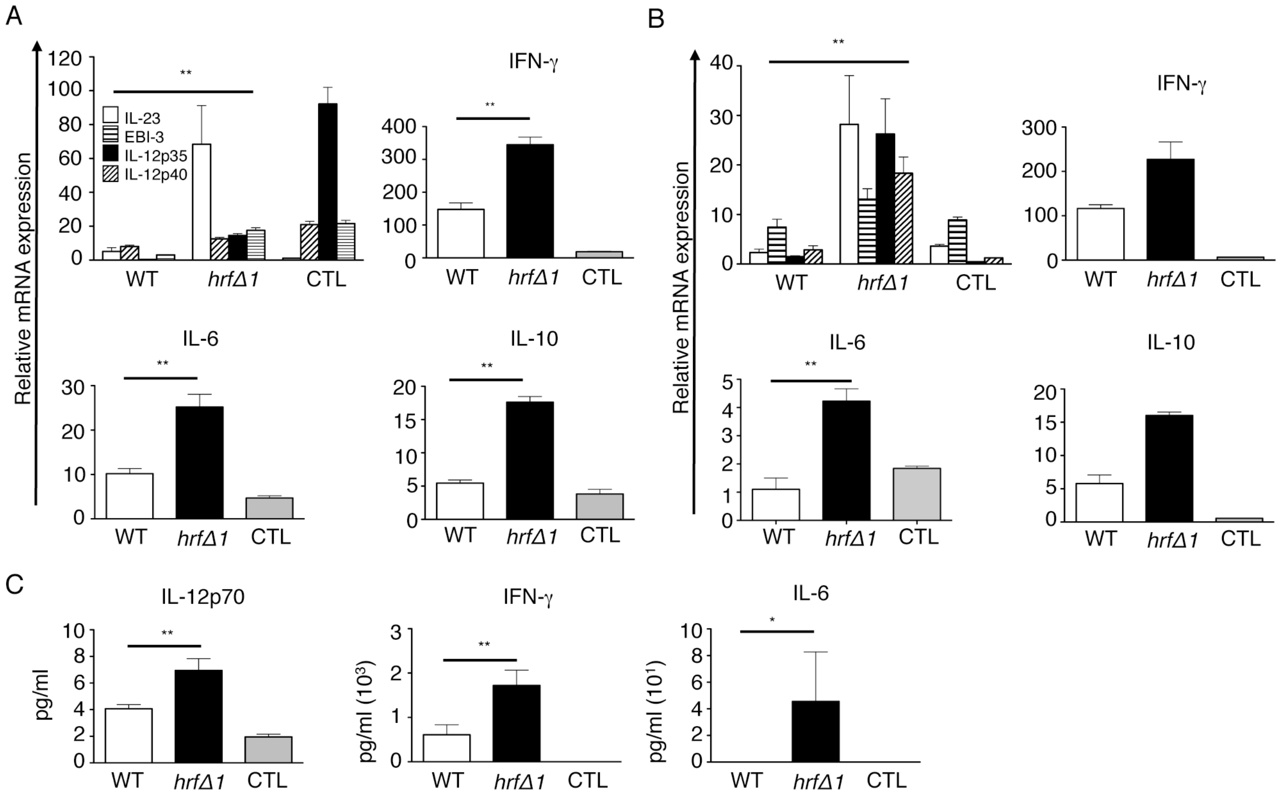

Supplementary figure 3

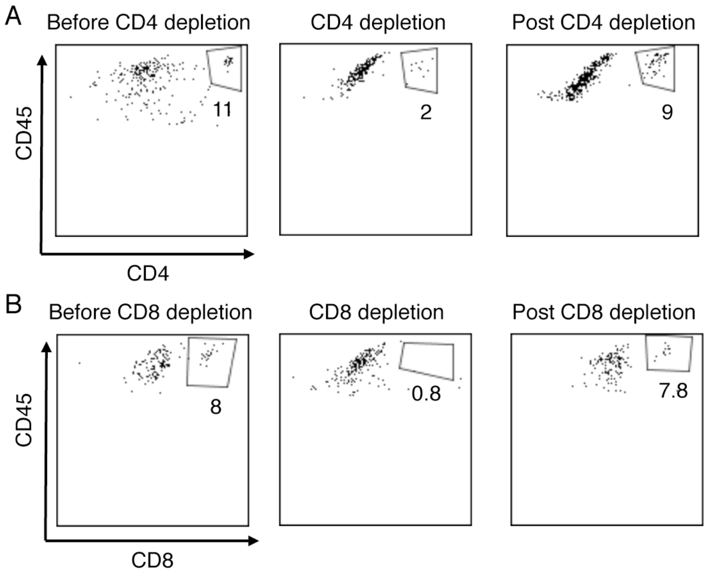

Supplementary figure 4

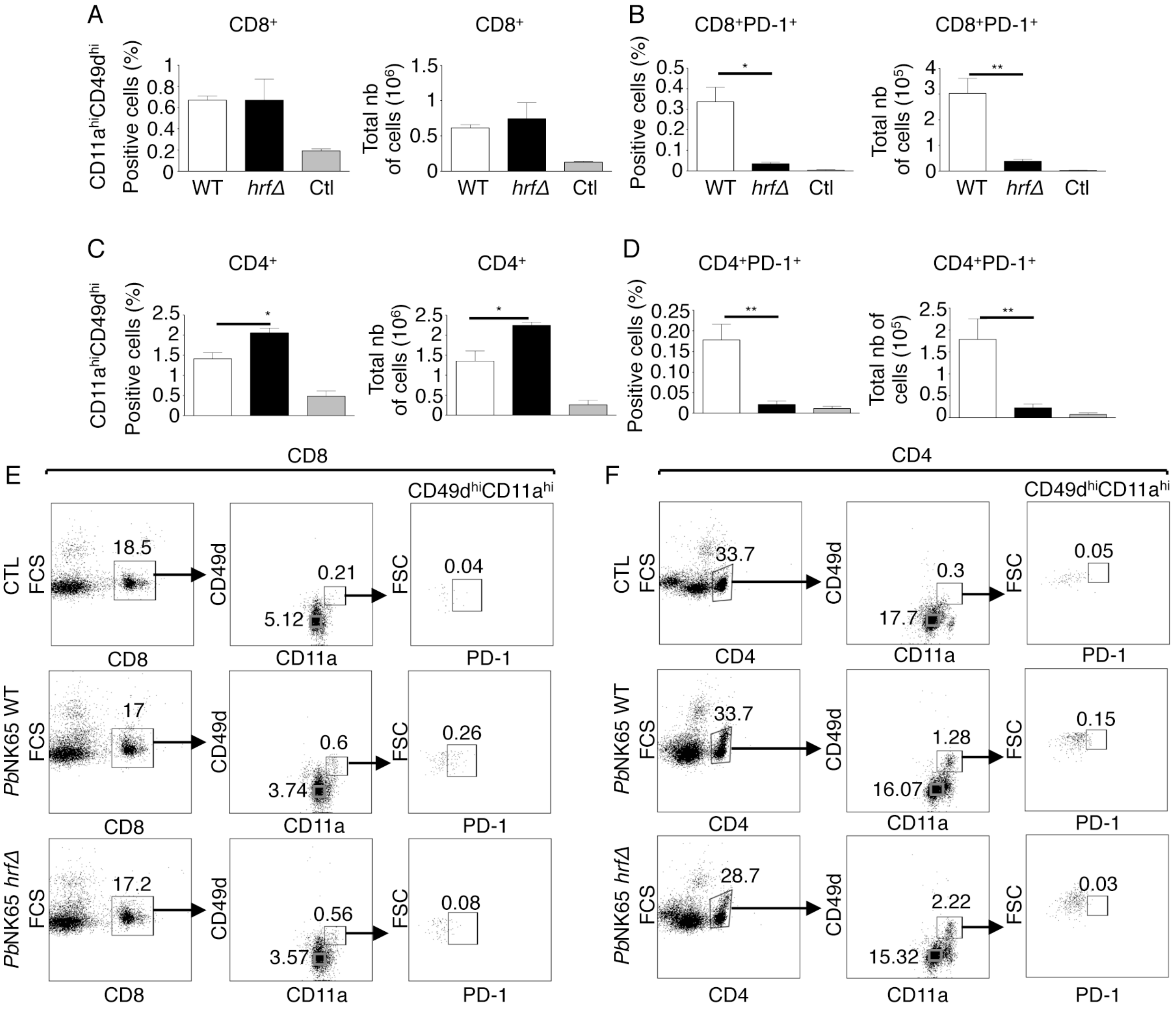

Supplementary figure 5

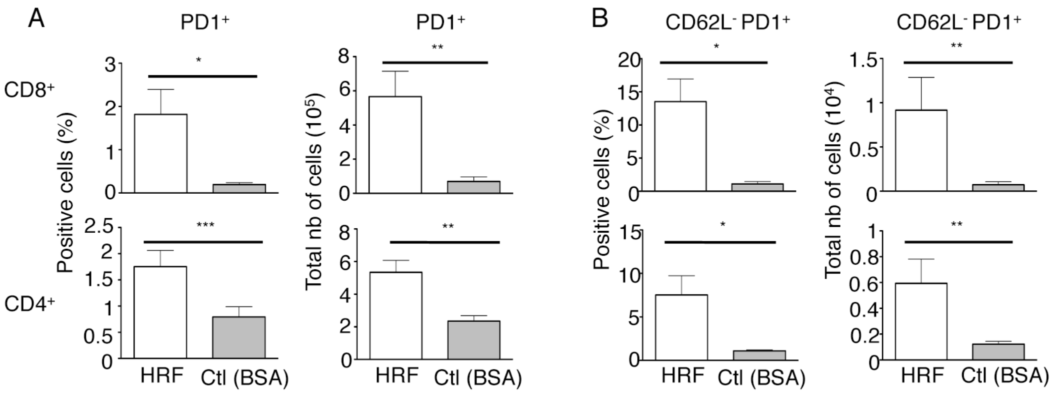

Supplementary figure 6

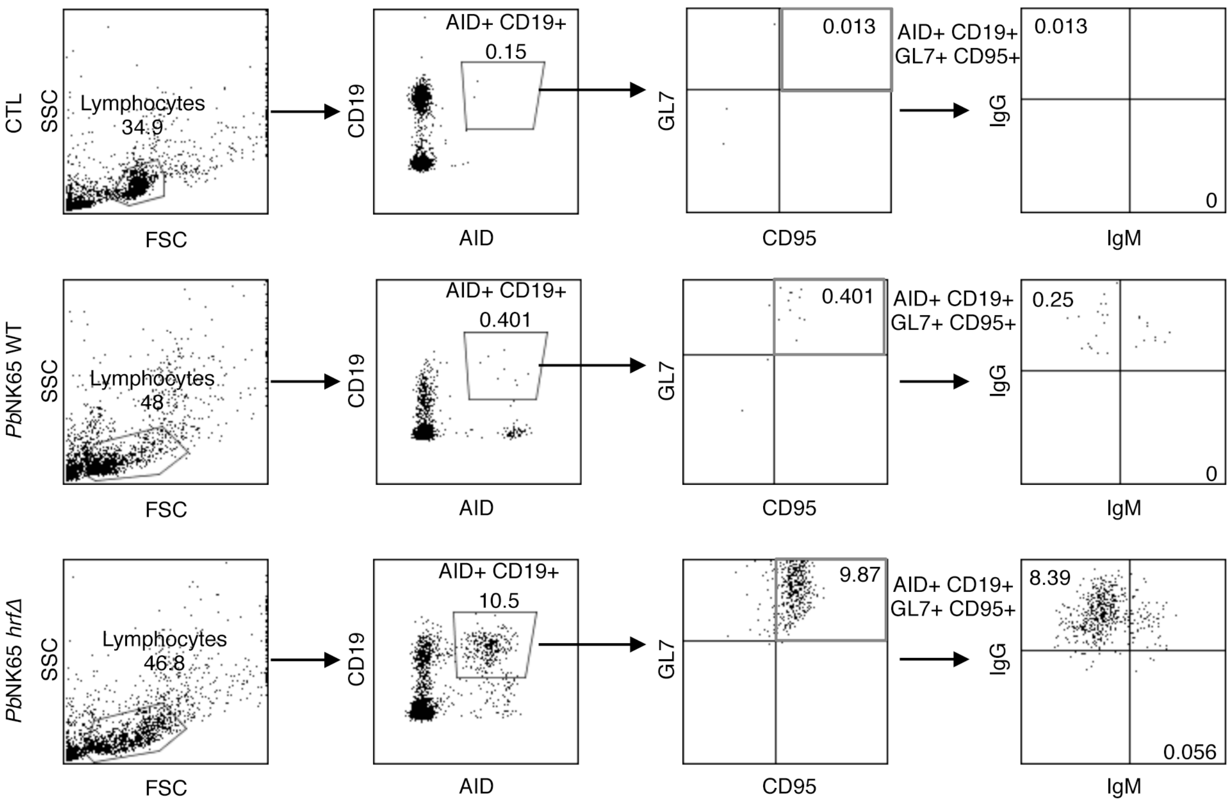

Supplement: Supplementary file 1 — Supplementary information [file 41598_2017_9684_MOESM1_ESM.pdf]
